# A Novel Macroblock Level Rate Control Method for Stereo Video Coding

**DOI:** 10.1155/2014/136854

**Published:** 2014-03-04

**Authors:** Gaofeng Zhu, Mei Yu, Gangyi Jiang, Zongju Peng, Feng Shao, Fen Chen, Yo-Sung Ho

**Affiliations:** ^1^Faculty of Information Science and Engineering, Ningbo University, Ningbo 315211, China; ^2^Deptartment of Information and Communication, Gwangju Institute of Science and Technology, Gwangju 500-712, Republic of Korea

## Abstract

To compress stereo video effectively, this paper proposes a novel macroblock (MB) level rate control method based on binocular perception. A binocular just-notification difference (BJND) model based on the parallax matching is first used to describe binocular perception. Then, the proposed rate control method is performed in stereo video coding with four levels, namely, view level, group-of-pictures (GOP) level, frame level, and MB level. In the view level, different proportions of bitrates are allocated for the left and right views of stereo video according to the prestatistical rate allocation proportion. In the GOP level, the total number of bitrates allocated to each GOP is computed and the initial quantization parameter of each GOP is set. In the frame level, the target bits allocated to each frame are computed. In the MB level, visual perception factor, which is measured by the BJND value of MB, is used to adjust the MB level bit allocation, so that the rate control results in line with the human visual characteristics. Experimental results show that the proposed method can control the bitrate more accurately and get better subjective quality of stereo video, compared with other methods.

## 1. Introduction

Three-dimensional (3D) videos, including stereo video multiview video and, have currently been coming to the home through various approaches, such as Blu-Ray disc, cable and satellite transmission, terrestrial broadcast, and streaming and download through Internet [1]. Moreover, 3D video technologies have been gradually matured to be moved into mobile platforms, for example, 3D mobile phone and mobile 3D television [2]. However, there are many crucial technologies that need further research for stereo video coding. Rate control is a key issue in stereo/multiview video coding and transmission.

Lu et al. [3] proposed a rate control scheme for multiview video coding (MVC), on the basis of the new quantitative measure for stereo video quality. And the macroblock (MB) quantization parameters (QPs) are modified according to the QP of the neighboring MB on the purpose of eliminating block effect in stereo video. Shao et al. proposed a rate control method for asymmetric stereo video coding [4]. They use a fixed threshold to quantize binocular psychovisual redundancy and establish the relationship between distortion and quantization for asymmetric stereo video coding. Zheng et al. [5] presented a rate control algorithm for MVC by analyzing the rate allocation proportion among different types of views, and the frame complexity was used to regulate the target bit for each frame. Liu et al. [6] presented a rate control technique for multiview video plus depth (MVD) based 3D video coding, and an image-stitching method was utilized to simultaneously encode video and depth. While these rate control algorithms in stereo/multiview video coding made useful explorations, however, these algorithms were only designed to frame layer bit allocation. In order to more finely rate control, it is necessary to develop the rate control techniques on the MB layer.

Moreover, the above algorithms did not take into account human visual characteristics well. Due to physiological and psychological mechanisms of human visual system (HVS), human eyes do not detect all of image or video distortion [7]. Thus, the concept of just-noticeable difference (JND) has played an important role in understanding HVS [8, 9]. JND implies a visibility threshold in which human eyes can perceive changes in image values and depends on the luminance and contrast of local image region. Liu et al. [10] utilized a JND model to separate edge and textured regions of the image. Recently, several studies had concentrated on constructing visibility threshold for 3D image/video. Based on modeling and incorporating the binocular combination of injected noises, luminance masking, and contrast masking, binocular JND (BJND) was reported as the first model to measure the perceptible distortion of stereo images [11]. It was empirically demonstrated that human cannot realize distortion in stereo image if the distortion in one view image is less than the BJND value.

In this paper, we propose a novel MB level rate control method for stereo video coding based on visual perception. The proposed rate control method is performed in stereo video coding with four levels, namely, view level, group-of-pictures (GOP) level, frame level, and MB level. The BJND model based on the parallax matching is first exploited. Then, visual perception factor, designed based on BJND, is used to adjust the MB level bit allocation. So the rate control results in line with human visual characteristics. The rest of this paper is organized as follows.  Section 2 describes the BJND model and Section 3 describes the proposed method.  Section 4 shows experimental results, and finally, a conclusion is given in Section 5.

## 2. The BJND Model

To account for the mutual effect on the minimum distortion in one view that evokes perceptual differences in stereo image, the BJND model was proposed by Zhao et al. and measured with a set of psychophysical stimuli [11]. It has been demonstrated that the BJND is binocular combination of injected noises, luminance masking, and contrast masking in binocular viewing.

In [11], Zhao et al. did not take into account the disparity between left and right views of stereo image in their experiments, and they obtained BJND values under assumption about that the disparities between left and right views were zero. This assumption is not reasonable since in most cases the disparity of stereo image is not zero. In practice, binocular disparity is attributed to the primary visual cortical areas, and it is one of the most important stereo cues. Stereo matching itself has been a difficult task in computer vision, and a comprehensive review of stereo matching is given in [12]. Here, a state-of-the-art stereo matching technique in [13] is adopted.

To improve Zhao's model, a modified BJND model is presented with the consideration of binocular disparity. Given the left and right views of stereo image with the disparity image corresponding to left view, by incorporating the models of the binocular combination of injected noises, luminance, and contrast masking effects, a BJND at the right view is defined as follows:

(1) BJNDr(i,j,d) =BJNDr(bgl(i+d,j),ehl(i+d,j),nl(i+d,j)) =TC(bgl(i+d,j),ehl(i+d,j))  ×(1−(nl(i+d,j)TC(bgl(i+d,j),ehl(i+d,j)))λ)1/λ,

where *i* and *j* are the pixel coordinates, *d* is the disparity at the position of (*i*, *j*) in right view, and*T*_*C*_ is a contrast masking threshold. Note that BJND_*r*_ is dependent on the background luminance level bg_*l*_, the edge height eh_*l*_, and the noise amplitude *n*_*l*_ of the corresponding pixel position in the left view. Here, the original sequence is used, so there is no noise in the left view; that is, *n*_*l*_ = 0.

*T*_*C*_ is defined by

(2) $$\begin{array}{c} T_{C}\left(\text{b}{\text{g}}_{l},\text{e}{\text{h}}_{l}\right)=A_{\text{limit}}\left(\text{b}{\text{g}}_{l}\right)+K\left(\text{b}{\text{g}}_{l}\right)\cdot \text{e}{\text{h}}_{l}, \end{array}$$

where *A*_limit_ ( ) is a luminance masking function and *K* ( ) is a fitting function.

*A*_limit_(bg_*l*_) and bg_*l*_ are calculated in

(3) Alimit(bgl)={0.0027·(bgl2−96·bgl)+8,if  0≤bgl<48,0.0001·(bgl2−32·bgl)+1.7,if  48≤bgl≤255,bg(x,y)=132∑i=0 i=5∑j=0j=5I(x−3+i,y−3+j)·B(i,j).

The fitting function *K*(bg_*l*_) of bg_*l*_ is defined as

(4) K(bgl)=−10−6·(0.7·bgl2+32·bgl)+0.07,bgl∈[0,255].

The edge height eh_*l*_ is given by

(5) $$\begin{array}{c} \text{e}{\text{h}}_{l}\left(i,j\right)=\sqrt{E_{H}^{2}\left(i,j\right)+E_{V}^{2}\left(i,j\right)}, \end{array}$$

where *E*_*k*_(*i*, *j*) is 5 × 5 Sobel operator and the formula is as follows:

(6)Ek(i,j)=124∑h=1 5∑v=15p(i−3+h,j−3+v)∗Gk(h,v),k=H,V,(7)GH=[−1−2021−2−3032−3−5053−2−3032−1−2021],GV=[123212353200000−2−3−5−3−2−1−2−3−2−1].

## 3. The Proposed BJND-Based Rate Control Method

**Figure 1:**
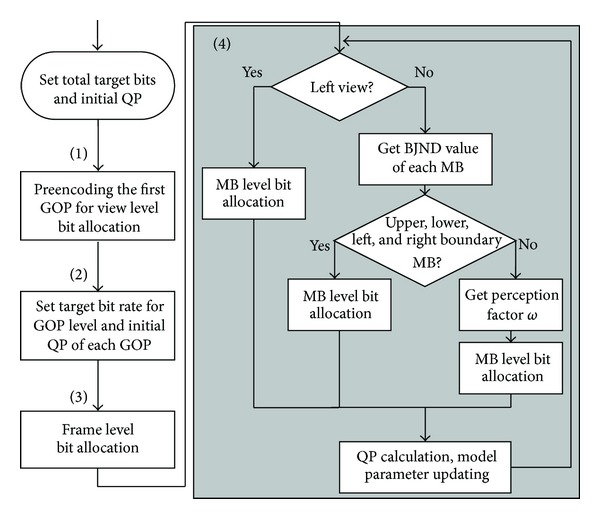
The framework of the proposed MB level rate control method for stereo video coding.

Here, we present a new rate control method for the stereo video based on the modified BJND. The framework of the proposed method is illustrated in Figure 1, with rate control in four levels, including view level, group-of-pictures (GOP) level, frame level, and MB level.

### 3.1. View Level Rate Control

**Figure 2:**
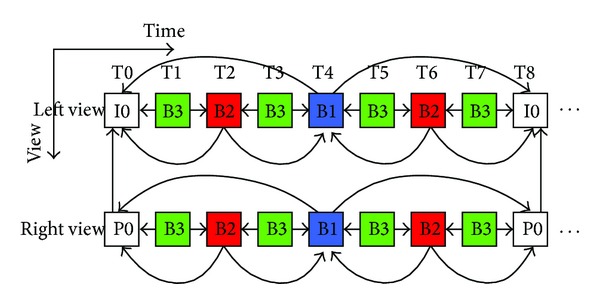
HBP coding structure of left view and right view.

Hierarchical B Pictures (HBP) coding structure in stereo video is shown in Figure 2. There are two views to be encoded. It is seen that there is no interview predication in left view, and right view is encoded with unidirectional interview prediction from the reconstructed left view.

In [14], we obtained the proportion between left view and right view according to preencoding the first GOP. The target bit rate for each view is computed by

(8) $$\begin{array}{c} T_{\text{view}}\left(k\right)=T_{\text{total}}\times w\left(k\right), \end{array}$$

where *T*_total_ denotes the total bit rate of left and right views. *w*(*k*) is bit allocation proportion for each view. *k* is the coding order index of view, and the value of *k* is 0 or 1, which represents the left view and right view, respectively; *T*_view_ (*k*) is target bit rate for the *k*th view.

### 3.2. GOP Level Rate Control

Within the left view or the right view, the total number of bits allocated to each GOP is computed and the initial QP of each GOP is set in GOP level rate control. The total number of bits allocated for the *i*th GOP is computed by

(9) $$\begin{array}{c} T_{r}\left(n_{i,0}\right)=\frac{T_{\text{view}}\left(k\right)}{F_{r}}\times N_{\text{gop}}-\left(\frac{B_{s}}{8}-B_{C}\left(n_{i-1,N_{\text{gop}}}\right)\right), \end{array}$$

where *F*_*r*_ is frame rate, *N*_gop_ denotes total number of frames in the current GOP, and *B*_*C*_(*n*_*i*−1,*N*_gop__) is actual buffer occupancy after encoding the (*i* − 1)th GOP. The buffer occupancy should be kept at *B*_*s*_/8 after encoding each GOP. In the case of the constant bit rate (CBR), *T*_*r*_ is updated frame by frame as follows:

(10) $$\begin{array}{c} T_{r}\left(n_{i,j}\right)=T_{r}\left(n_{i,j-1}\right)-A\left(n_{i,j-1}\right), \end{array}$$

where *A*(*n*_*i*,*j*−1_) is the actual encoding bits for the (*j* − 1)th frame.

The initial QP of the first GOP is a predefined QP, denoted as QP_0_. As shown in Figure 2, I0 and B1 frames in the first GOP of the left view and P0 and B1 frames in the first GOP of the right view are encoded by QP_0_. The first frame QP in other GOP is computed by

(11) $$\begin{array}{c} \text{Q}{\text{P}}_{\text{st}}=\frac{\text{Su}{\text{m}}_{\text{BQP}}}{N_{b}}-1-\frac{8T_{r}\left(n_{i-1,N_{\text{gop}}}\right)}{T_{r}\left(n_{i,0}\right)}-\frac{N_{\text{gop}}}{15}, \end{array}$$

where *N*_*b*_ is total number of bits in encoding B frame in a GOP and Sum_BQP_ is the sum of QPs for all B frames in the previous GOP.

### 3.3. Frame Level Rate Control

Within each GOP, the target bits for each B frame are calculated according to the target buffer fullness level and the number of remaining bits in frame level rate control.

The actual buffer occupancy, *T*_buf_(*n*_*i*,*j*_), is computed as

(12) Tbuf(ni,j)=Tview(k)Fr +γ(Tbl(ni,j−1)−Tbl(ni,2)Nb−1−BC(ni,j)),

where *γ* is a constant and its typical value is 0.75 [15]. *Tbl*(*n*_*i*,*j*−1_) is the target buffer level of the *j*-1th frame in the *i*th GOP. *Tbl*(*n*_*i*,2_) is the initial value of target buffer level. *B*_*C*_(*n*_*i*,*j*_) is the actual buffer occupancy after coding the *j*th B frame in the *i*th GOP.

Meanwhile, the number of remaining bits should also be considered when the target bit is computed.

(13) $$\begin{array}{c} T_{\text{rem}}\left(n_{i,j}\right)=\frac{T_{r}\left(n_{i,j}\right)}{N_{b,r}\left(j-1\right)}, \end{array}$$

where *N*_*b*,*r*_(*j* − 1) is the number of the remaining B frames in the *i*th GOP.

The target bits, *T*(*n*_*i*,*j*_), are a weighted combination of *T*_buf_(*n*_*i*,*j*_) and *T*_rem_(*n*_*i*,*j*_) and represented by

(14) $$\begin{array}{c} T\left(n_{i,j}\right)=\beta \times T_{\text{rem}}\left(n_{i,j}\right)+\left(1-\beta \right)\times T_{\text{buf}}\left(n_{i,j}\right), \end{array}$$

where *β* is a constant and its typical value is 0.5 [15].

### 3.4. MB Level Rate Control

Let *T*_rb_*l*__(*n*_*i*,*j*_) and *N*denote the number of remaining bits for the all noncoded macroblock in the current frame and the number of MBs, respectively. Let *T*_mb_*l*__(*i*, *j*, *k*) and MAD(*i*, *j*, *k*) denote target bit rate and MAD value in the *k*th MB, respectively. Consider

(15) $$\begin{array}{c} T_{{\text{mb}}_{l}}\left(i,j,k\right)=T_{{\text{rb}}_{l}}\left(n_{i,j}\right)\times \frac{\text{MA}{\text{D}}_{l}^{2}\left(i,j,k\right)}{\sum_{p=k}^{N}\text{MA}{\text{D}}_{l}^{2}\left(i,j,p\right)}. \end{array}$$

With the current MB's BJND value and the average BJND value of removing the left, right, upper, and lower boundary MBs, the tolerable distortion degree, *μ*(*i*, *j*, *k*), of the current MB in the whole frame is measured as follows:

(16) $$\begin{array}{c} \mu \left(i,j,k\right)=\frac{\text{BJN}{\text{D}}_{r}\left(i,j,k\right)}{\sum_{u=2}^{X-1}\sum_{v=2}^{Y-1}\text{BJN}{\text{D}}_{r}\left(i,j,u,v\right)}, \end{array}$$

where *X* and *Y* denote the number of MBs in a row and a column for each frame, respectively. The fluctuation of *μ*(*i*, *j*, *k*) is large, thus, it is normalized and then added with 0.5 so as to get the perception factor weighting *ω*(*i*, *j*, *k*), which is denote by

(17) $$\begin{array}{c} \omega \left(i,j,k\right)=\frac{\mu \left(i,j,k\right)-\mu_{\min}}{\mu_{\max}-\mu_{\min}}+0.5,\\ \mu_{\min}=\min\left\{\overset{X-1}{\underset{u=2\;}{\sum }}\overset{Y-1}{\underset{v=2}{\sum }}\text{BJN}{\text{D}}_{r}\left(i,j,u,v\right)\right\},\\ \mu_{\max}=\max\left\{\overset{X-1}{\underset{u=2\;}{\sum }}\overset{Y-1}{\underset{v=2}{\sum }}\text{BJN}{\text{D}}_{r}\left(i,j,u,v\right)\right\}, \end{array}$$

where min{ } indicates the minimum function and max{ } indicates the maximum function.

The perception factor *ω*(*i*, *j*, *k*) is used to adjust the target bit allocation for the MB level. The larger BJND value represents more sensitive to distortion for human eye; that is, the value of *ω*(*i*, *j*, *k*) is larger, so the MB should be allocated more bits. Conversely, the MB with smaller BJND value should be allocated smaller bits. Consider

(18) Tmbr(i,j,k)=Trbr(ni,j)×ω(i,j,k)×MADr2(i,j,k)∑p=kNMADr2(i,j,p).

Based on the allocated target bits, the quantization step size can be computed by the quadratic R-Q model:

(19) $$\begin{array}{c} T_{\text{mb}}\left(i,j,k\right)=\left(\frac{X_{1}}{Q_{\text{step}}^{2}}+\frac{X_{2}}{Q_{\text{step}}}\right)\times \text{MAD}\left(i,j,k\right), \end{array}$$

where *Q*_step_ is the quantization step size of the current MB. *X*_1_ and *X*_2_ are the model parameters, which need to be updated using a linear regressive technique.

Furthermore, QP of the current MB can be computed by

(20) $$\begin{array}{c} \text{QP}=6\times \text{lo}{\text{g}}_{2}\left(Q_{\text{step}}\right)+4. \end{array}$$

## 4. Experimental Results and Discussions

In order to evaluate the performance of the proposed MB level rate control method for stereo video coding based on visual perception, six representative stereo video sequences with 1024 × 768 spatial resolution, including *BookArrival*, *AltMoabit*, *DoorFlowers*, *LeavingLaptop*, *Newspaper*, and *Kendo*, are used in the experiments. *BookArrival*, *AltMoabit*, *DoorFlowers*, and *LeavingLaptop* are provided by Fraunhofer HHI [16]. *Newspaper* is provided by Gwangju Institute of Science and Technology [17]. *Kendo* sequence is captured by Nagoya University with the moved camera array [18].

In the experiments, we use the revised MVC software JMVC7.0 to implement the rate control methods. The test conditions of the six sequences are shown in Table 1. Two middle views (view 9 and view 10) for *BookArrival*, *DoorFlowers*, and *LeavingLaptop*, two middle views (view 5 and view 6) for *AltMoabit *and *Newspaper*, the two middle views (view 4 and view 5) for *Kendo* are used to simulate the left and right views of stereo video in Figure 2.

**Figure 3:**
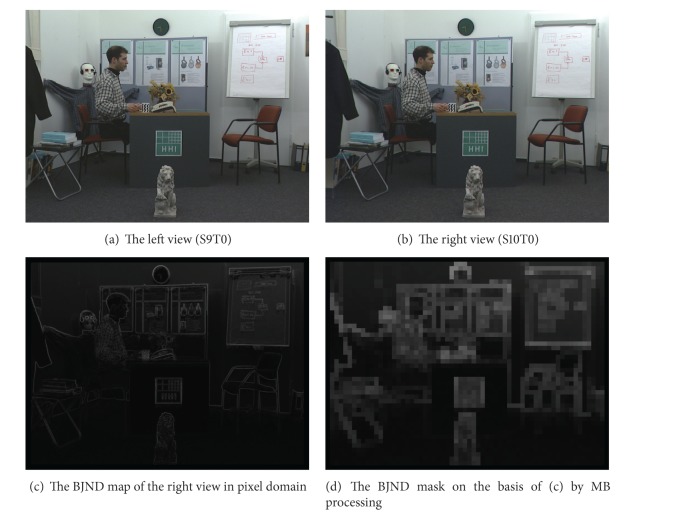
The BJND of the* BookArrival* sequence.

**Figure 4:**
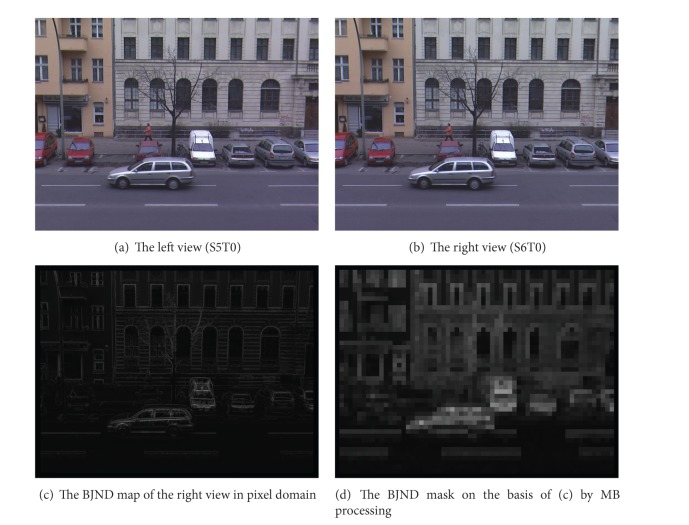
The BJND of* AltMoabit* sequence.

In order to avoid the influence of BJND results caused by occlusion and exposure in the left and right views of stereo video, the left, right, upper, and lower MBs in each frame of the right view sequence are not processed. And the BJND results of *BookArrival* and *AltMoabit* are given in this paper, as shown in Figures 3 and 4, respectively. Figures 3(a) and 3(b) show the original left and the right view images.  Figure 3(c) is the BJND map of the right view in pixel domain, and Figure 3(d) is the BJND mask on the basis of Figure 3(c) by MB processing. In the same way, Figure 4 shows the similar results.

### 4.1. Rate Control Accuracy in Stereo Video Coding

Under the test conditions shown in Table 1, three methods, Zheng's in [5], SMBRC, and the proposed method, are compared. SMBRC denotes the stereo video MB level rate control method; that is, the MB level monoscopic video JVT-G012 algorithm is extended to stereo video coding and implemented in JMVC.

The detailed average control accuracies of three methods are shown in Table 2. In Table 2, the target bitrate and the actually controlled bitrate are the average value of two views. Rate control error ratio, *E*_RC_, is used to measure the accuracy of the bitrate estimation and defined by

(21) $$\begin{array}{c} E_{\text{RC}}=\frac{\left|R_{\text{actual}}-R_{\text{target}}\right|}{R_{\text{target}}}\times 100\%, \end{array}$$

where *R*_target_ and *R*_actual_ denote the target bitrate and the actual coding bitrate, respectively.

Zheng's method is a frame-level rate control method, while the proposed method and SMBRC are MB level rate control scheme, so they have better control accuracy. This can be evidently seen from Table 2. Table 2 also indicates that the absolute inaccuracy of the proposed stereo video rate control method is within 0.192%. It is obvious that the proposed method can more precisely control the bitrate in stereo video coding.

### 4.2. Rate Distortion Performance Comparison

**Figure 5:**
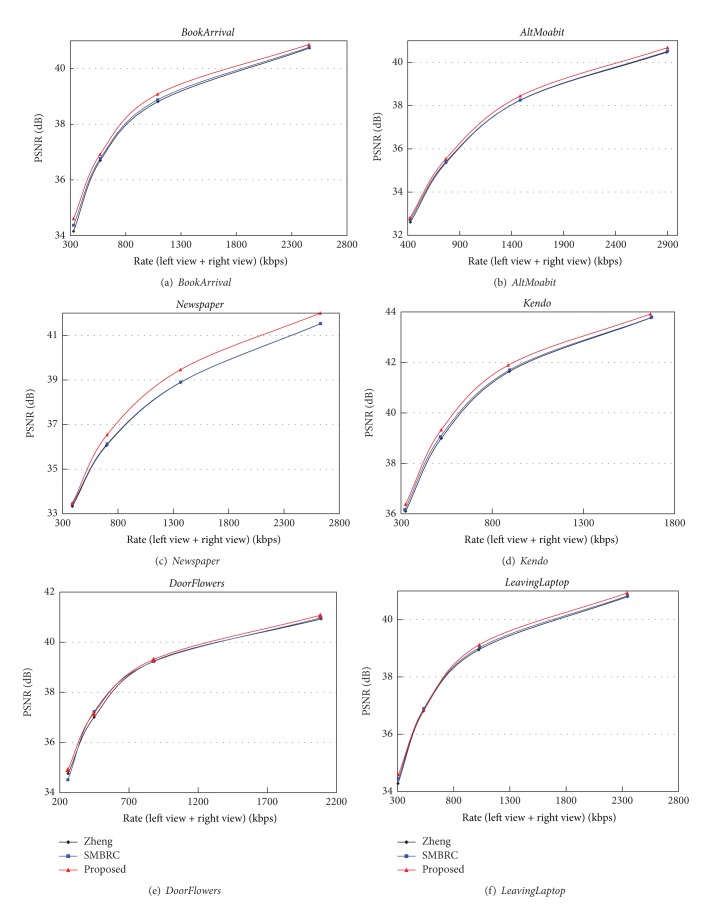
The R-D performance comparisons of three methods.

Figure 5 shows the rate-distortion (R-D) performance comparison results of the three methods. In Figure 5, the average bitrate is the average value of two views, and the peak signal-to-noise ratio (PSNR) value is also the average value of two views. It is clear that, compared with Zheng's and SMBRC methods, the proposed method can achieve the better R-D performance under different target bitrates since more accurate binocular perception information is used in the proposed method.

### 4.3. Subjective Quality Comparison

**Figure 6:**
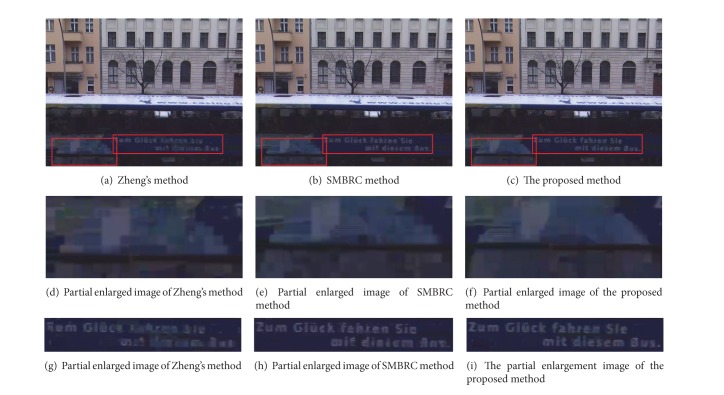
The subjective image comparison among three methods of *AltMoabit* sequence (QP = 37, the 52th frame in the view 6).

Different sequences possess different motion and scene characteristics; therefore the video frames which can best reflect the differences in the subjective quality are selected in accordance with the characteristics of each sequence. For the outdoor scene *AltMoabit* sequence, we select the 52th frame in view 6 as an example, shown in Figure 6. The bus and the words on the bus in the image are the areas of human visual attention in Figure 6, and more bits are allocated for these areas under the visual perception factor based on BJND. Therefore the subjective quality of the proposed method is better than the other methods.

**Figure 7:**
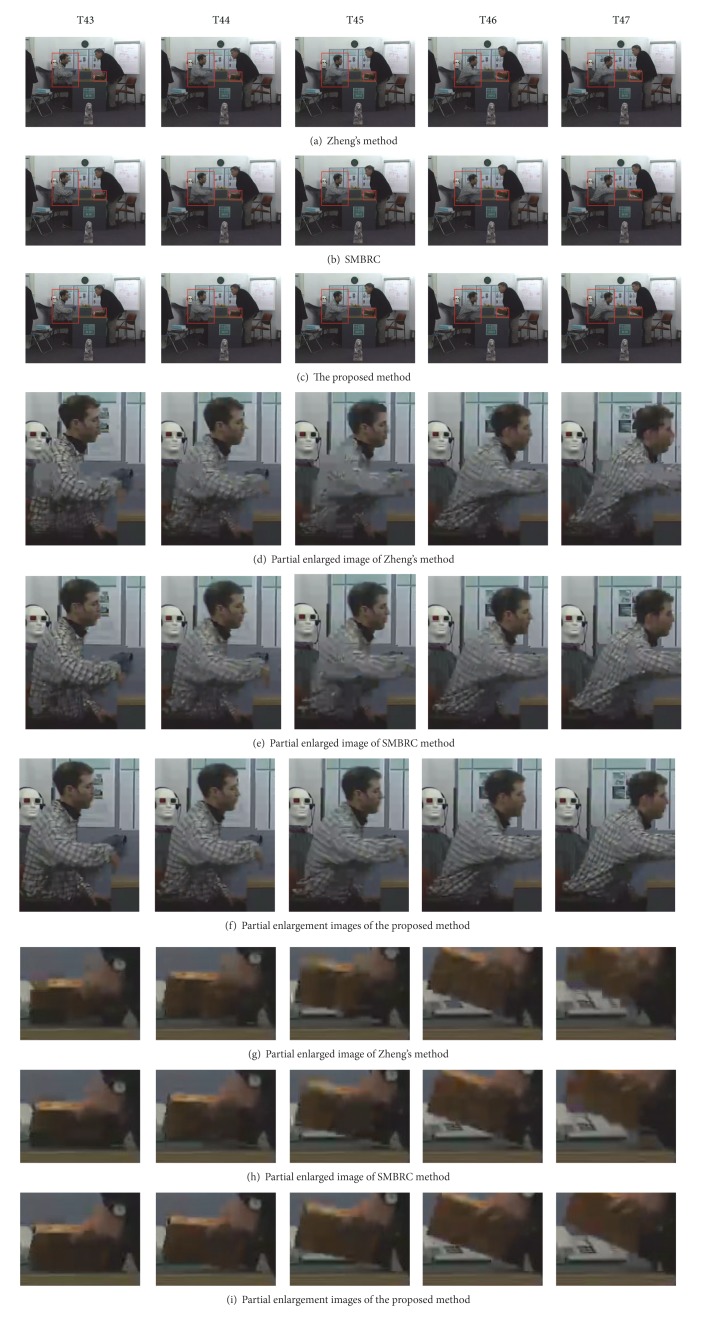
The subjective image comparison for consecutive frames of *LeavingLaptop* sequence (QP = 37, the 43th frame to 47th frame in view 10).

In order to eliminate the limitation of only one video frame, we select the 43rd frame to 47th frame for the indoor scene *LeavingLaptop* sequence, shown in Figure 7. The details of the human body and head and the book are allocated more bitrates, so the subjective quality of the proposed method is better than the other methods. Moreover, for quantization parameter selection, if we select a small value QP, the subjective quality of the three methods is too good to reflect the difference. Hence, QP is set to 37.

## 5. Conclusion

A novel macroblock (MB) level rate control method for stereo video is proposed in this paper. The proposed method is performed with four levels, namely, view level, group-of-picture (GOP) level, frame level, and MB level. In the view level, different proportions of bitrates are allocated for left view and right view of stereo video according to the prestatistical rate allocation proportion. In the GOP level, the total number of bitrates allocated to each GOP is computed and the initial quantization parameter of each GOP is set. In the frame level, the target bits allocated to each frame are computed. In the MB level, the binocular just-noticeable difference (BJND) model based on the parallax matching is first given. Then, visual perception factor, which is measured by the ratio between the BJND value of the current MB and the average BJND value of removing the left, right, upper, and lower boundary MBs, is used to adjust the MB level bit allocation. Experimental results show that the proposed method can control the bitrate more accurately and the average of rate control error is only about 0.19%. Compared with other schemes, the proposed method gets better rate-distortion performance and subjective quality of stereo images.

## Figures and Tables

**Table 1:** Test conditions.

Frame rate	15	Channel type	CBR
GOP length	8	Search mode	Fast search
Search range	32	Frame's no.	97
Reference frames	2	Symbol mode	CABAC

**Table 2:** The detailed control accuracies of the three schemes.

Sequence	Target bitrate (kbps)	Actual bitrate (Kbps)			*E*_RC_ (%)		
		Zheng	SMBRC	Proposed	Zheng	SMBRC	Proposed
*BookArrival *	2454.588	2461.882	2457.641	2456.328	0.297	0.124	0.071
	1087.928	1091.650	1089.203	1088.316	0.342	0.117	0.036
	569.213	571.351	571.052	569.973	0.376	0.323	0.133
	328.962	329.536	329.185	329.137	0.174	0.068	0.053

*AltMoabit *	2898.506	2895.074	2902.553	2901.796	0.118	0.140	0.113
	1482.783	1481.250	1485.130	1482.949	0.103	0.158	0.011
	766.879	771.416	768.551	767.346	0.592	0.218	0.061
	422.260	425.840	424.130	422.078	0.848	0.443	0.043

*Newspaper *	2624.359	2629.377	2630.822	2626.997	0.191	0.246	0.101
	1363.146	1365.172	1366.715	1365.219	0.149	0.262	0.152
	700.768	697.747	703.104	702.132	0.431	0.333	0.195
	387.959	389.030	388.711	388.285	0.276	0.194	0.084

*Kendo *	1664.009	1669.532	1673.897	1666.769	0.332	0.594	0.166
	889.142	894.546	895.593	887.494	0.608	0.726	0.185
	518.328	521.145	515.668	520.191	0.543	0.513	0.359
	323.106	325.386	320.659	325.120	0.706	0.757	0.623

*DoorFlowers *	2081.433	2088.817	2085.565	2082.963	0.355	0.199	0.073
	878.540	882.652	879.054	877.835	0.468	0.059	0.080
	448.642	448.616	448.214	444.370	0.006	0.095	0.952
	259.811	259.945	259.116	258.028	0.051	0.268	0.686

*LeavingLaptop *	2343.246	2347.945	2347.679	2344.263	0.201	0.189	0.043
	1029.631	1023.200	1031.212	1027.668	0.625	0.154	0.191
	534.344	531.283	533.795	533.878	0.573	0.103	0.087
	307.944	304.321	307.543	308.274	1.176	0.130	0.107

Average					0.398	0.267	0.192
